# Curcumin Nanoparticles and Their Cytotoxicity in Docetaxel-Resistant Castration-Resistant Prostate Cancer Cells

**DOI:** 10.3390/biomedicines8080253

**Published:** 2020-07-30

**Authors:** Irin Tanaudommongkon, Asama Tanaudommongkon, Priyanka Prathipati, Joey Trieu Nguyen, Evan T. Keller, Xiaowei Dong

**Affiliations:** 1 Department of Pharmaceutical Sciences, University of North Texas Health Science Center, Fort Worth, TX 76107, USA; it0046@live.unthsc.edu (I.T.); at0371@live.unthsc.edu (A.T.); priyankaprathipati@gmail.com (P.P.); jhn0043@my.unthsc.edu (J.T.N.); 2 Department of Urology and Biointerfaces Institute, University of Michigan, Ann Arbor, MI 48109, USA; etkeller@umich.edu

**Keywords:** sensitive and resistant prostate cancer cells, cytotoxicity, therapeutic agents, lipid-based nanoparticles, P-glycoprotein

## Abstract

Most prostate cancer patients develop resistance to anti-androgen therapy. This is referred to as castration-resistant prostate cancer (CRPC). Docetaxel (DTX) is the mainstay treatment against CRPC. However, over time patients eventually develop DTX resistance, which is the cause of the cancer-related mortality. Curcumin (CUR) as a natural compound has been shown to have very broad pharmacological activities, e.g., anti-inflammatory and antioxidant properties. However, CUR is very hydrophobic. The objective of this study was to develop CUR nanoparticles (NPs) and evaluate their cytotoxicity in DTX-resistant CRPC cells for the treatment of DTX-resistant CRPC. We tested solubility of CUR in different lipids and surfactants. Finally, Miglyol 812 and D-alpha-tocopheryl poly (ethylene glycol) succinate 1000 (TPGS) were chosen to prepare lipid-based NPs for CUR. We fully characterized CUR NPs that had particle size < 150 nm, high drug loading (7.5%), and entrapment efficiency (90%). Moreover, the CUR NPs were successfully lyophilized without using cryoprotectants. We tested the cytotoxicity of blank NPs, free CUR, and CUR NPs in sensitive DU145 and PC3 cells as well as their matching docetaxel-resistant cells. Cytotoxicity studies showed that blank NPs were very safe for all tested prostate cancer cell lines. Free CUR overcame the resistance in PC3 cells, but not in DU145 cells. In contrast, CUR NPs significantly increased CUR potency in all tested cell lines. Importantly, CUR NPs completely restored CUR potency in both resistant DU145 and PC3 cells. These results demonstrate that the CUR NPs have potential to overcome DTX resistance in CRPC.

## 1. Introduction

Prostate cancer (PCa) is the second-most-frequent cancer diagnosis in men and the fifth leading cause of cancer death worldwide [1]. Anti-androgen is the first line treatment for PCa. However, most patients will develop resistance to anti-androgen therapy and high metastases, which is referred to as castration-resistant prostate cancer (CRPC) [2]. Docetaxel (DTX) was approved by the Food and Drug Administration (FDA) as the mainstay treatment against CRPC and has been shown to improve the survival rate of men with metastatic CRPC [3]. However, over time patients eventually develop DTX resistance, which is the cause of the cancer-related mortality. Multiple mechanisms exist for drug resistance and a drug is not confronted by a single mechanism. One well-known mechanism of drug resistance is overexpression of P-glycoprotein (P-gp), a transporter that can efflux the drugs out of cells [4]. Fairly compelling data showed the correlation between P-gp overexpression and poor clinical outcomes in many cancers, including PCa [5,6]. Indeed, overexpression of P-gp is the main cause of resistance in docetaxel-resistant DU145 cells [7]. To date, the use of P-gp inhibitors to enhance efficacy in drug-resistant cancers has not been successful in clinical trials [8]. Because of the lack of specificity, P-gp inhibitors also inhibit P-gp in normal cells, leading to significant side effects. Even though new cytotoxicity agents, e.g., cabazitaxel, were developed to avoid P-gp recognition, tumor heterogeneity still generates drug resistance such as up-regulation of apoptosis genes and microtubulin alterations in CRPC [7]. Thus, a new therapeutic agent is needed to overcome multiple resistance mechanisms in CRPC.

Curcumin (CUR) is a bioactive compound extracted from the rhizome of the *Curcuma longa* plant. It has a low molecular weight and is a yellow, lipophilic polyphenolic compound. Due to a wide range of biological and pharmacological activities, CUR contains anti-inflammatory and antioxidant properties, and has been studied as a chemopreventative and chemotherapeutic agent [9]. CUR is also involved in cell cycle control and stimulation of apoptosis. It modulates autophagy and has inhibitory effects on tumor angiogenesis and metastasis in numerous human cancers [10,11]. Combination of taxanes and CUR has demonstrated synergistic cytotoxicity in sensitive PC3 cells [12]. However, the influence of CUR for DTX-resistant CRPC is unknown.

The application of CUR in clinical trials has been limited due to hydrophobicity, low water solubility, instability, and poor pharmacokinetics in spite of its superior properties [13,14,15]. Nanoparticles (NPs) were designed to deliver CUR in order to enhance its efficacy in cancers. NPs have the potential to protect drugs from degradation, enhance drug stability, control the drug release, alter and improve the pharmacokinetic and pharmacodynamic properties, and decrease toxicity [16,17]. Lipid-based NPs were chosen due to the advantages of being able to pass through leaky and hyperpermeable tumor vasculature and accumulate in the tumor vicinity by utilizing the enhanced permeability and retention (EPR) effect [18]. Importantly, they have been explored to overcome drug resistance in cancers. Some surfactants have been shown the potential to inhibit P-gp function. For example, d-alpha-tocopheryl poly (ethylene glycol) succinate 1000 (TPGS) is a vitamin E derivative and has been approved by the FDA as a safe pharmaceutical excipient used in many formulations. TPGS inhibits drug efflux through an allosteric modulation of P-gp [19].

The aim of this study was to develop CUR NPs and evaluate their cytotoxicity in DTX-resistant CRPC cells for the treatment of DTX-resistant CRPC. We used Miglyol 812 and TPGS to prepare NPs that encapsulated CUR, so called CUR MT NPs. We fully characterized the NPs in terms of particle size, zeta potential, polydispersity index (P.I.), drug entrapment efficiency (EE), drug loading (DL), differential scanning calorimetry (DSC) analysis, stability studies, and in vitro release study. Moreover, we tested the cytotoxicity of blank MT NPs, free CUR and CUR MT NPs in sensitive DU145 and PC3 cells and their matching DTX-resistant cells.

## 2. Materials and Methods

### 2.1. Chemicals and Reagents

CUR was purchased from Adipogen Corporation (San Diego, CA, USA). Phosphate buffer saline was purchased from American Type Culture Collection (ATCC) (Manassas, VA, USA). TPGS, Kollisolv MCT70, Poloxamer 188, Tween 20, and Tween 80 were obtained as gifts from BASF (Ludwigshafen, Germany). Miglyol 812, Miglyol 829, Imwitor 491, Imwitor 900K, and Imwitor 960 were obtained as gifts from Cremer (Eatontown, NJ, USA). Labrafac, Compritol 888, Labrasol, and Gelucire 44/14 were obtained as gifts from Gattefossé (Saint Priest Cedex, France). Microcon Y-100 with molecular cutoff 100 kDa was purchased from Millipore (Bedford, MA, USA). Ethanol USP grade was purchased from Pharmco-AAPER (Brookfield, CT, USA). Phosphoric acid was purchased from Sigma-Aldrich (St. Louis, MO, USA). Acetonitrile and methanol (HPLC grade) were purchased from Fisher Scientific (Pittsburgh, PA, USA). The sensitive prostate cancer cell lines, PC3 and DU145, were purchased from ATCC. PC3 and DU145 taxane-sensitive and -resistant cells were previously described [20]. Cells were maintained in Roswell Park Memorial Institute (RPMI) 1640 supplemented with 10% fetal bovine serum (FBS) and 1% penicillin–streptomycin. Cells were cultured at 37 °C in a humidified incubator with 5% CO_2_ and maintained in exponential growth phase by periodic subcultivation. Resistant cell lines were maintained with the addition of 5 nM docetaxel in the culture medium. Identification of the cells were confirmed using short tandem repeat analysis.

### 2.2. HPLC Analysis for Curcumin

The concentration of CUR was quantified by high-performance liquid chromatography (HPLC) using a Waters Alliance HPLC system and an Inertsil ODS-3 column (4.6 mm × 150 mm, 5 µm particle size, GL Sciences Inc., Torrance, CA, USA). The chromatography conditions were as follows: the mobile phase consisted of 0.1% orthophosphoric acid and acetonitrile (40:60, *v*/*v*) at a flow rate of 1.0 mL/min, and the effluent was detected at 420 nm. The injection volume was 30 µL, and the analysis time was 6.5 min per sample. The retention time of CUR was 5.7 min. The HPLC method was validated for the linear range, limitation of detection, accuracy, and precision. For the CUR standard curve, CUR was dissolved in methanol. The curve was found to be linear in the concentration range 0.1–50 µg/mL and r^2^ = 0.998.

### 2.3. Screening of Oils and Surfactants

The solubility of CUR was estimated in oils (Miglyol 812, Miglyol 829, Imwitor 491, Kollisolv MCT70, Labrafac, Imwitor 900K, Imwitor 960, and Compritol 888) and surfactants (Tween 20, Tween 80, Labrasol, Poloxamer 188, TPGS, and Gelucire 44/14). Oils and surfactants were accurately weighed into glass vials. An excess amount of CUR was added into glass vials containing an excipient. At each addition, the vials were stirred on a hot plate maintained at 65 °C for 3 min. The drug solubility (drug/excipient, *w*/*w*) was measured.

### 2.4. Preparation of Curcumin Nanoparticles

NPs were prepared by an emulsion method as reported elsewhere with some modification [20]. Miglyol 812 (4 mg) and TPGS (4 mg) were heated to 35 °C. Two milliliters of deionized (D.I.) water pre-heated at 65 °C was added into the mixture of melted oils and surfactants. The mixture was stirred for 20 min and then cooled down to room temperature. To prepare CUR MT NPs, 75 µL of 4 mg/mL of CUR dissolved in ethanol was directly added to the melted oil and surfactant; ethanol was removed by N_2_ stream and the NPs were prepared as described above.

### 2.5. Solubility of Curcumin in Water and the Cell Medium

The solubility of CUR was tested in D.I. water and the cell medium at 25 °C for 48 h, respectively. Briefly, excess CUR powders were added in each liquid. After 48 h, the sample was centrifuged at 14,000 rpm for 5 min. Then, the supernatant was transferred to an empty Eppendorf tube and centrifuged again at 14,000 rpm for 5 min. The same procedure was repeated one more time. The concentration of CUR in the final supernatant was determined using HPLC assay.

### 2.6. Particle Size and Zeta Potential Measurement

Particle size and size distribution of NPs were measured using a Delsa Nano C Particle Size Analyzer (Beckman Coulter Inc., Brea, CA, USA). Ten microliters of NPs were diluted with 1 mL of D.I. water to reach the density range required by the instrument, and particle size analysis was performed at 90° light scattering at 25 °C. PBS buffer (10 µL) was added for the measurement of zeta potential by using the Analyzer.

### 2.7. Morphology of CUR MT NPs

Five microliters of CUR MT NPs was applied to a carbon formvar-coated copper grid and stained with 2% aqueous uranyl acetate for 1 min. Grids were visualized with a Tecnai G^2^ spirit transmission electron microscopy (TEM) (ThermoFisher, Hillsboro, OR, USA) equipped with a LaB_6_ source at 120 kV using a Gatan Ultrascan CCD camera.

### 2.8. Determination of Drug Loading and Entrapment Efficiency

To quantify CUR in NPs, 1 part of CUR MT NPs was dissolved in 8 parts of methanol to measure DL%. The EE% was determined by separating free CUR and CUR MT NPs using Microcon Y-100 by centrifugation at 14,000 rpm at 4 °C, and then measuring CUR in the NPs. The DL% and EE% were calculated based on the following equations:

$$\mathrm{EE}\%=\frac{\mathrm{drug}\;\mathrm{entrapped}\;\mathrm{in}\;\mathrm{NPs}}{\mathrm{total}\;\mathrm{drug}\;\mathrm{added}\;\mathrm{into}\;\mathrm{NP}\;\mathrm{preparation}}\times 100\%\;\left(w|w\right)$$

$$\mathrm{DL}\%=\frac{\mathrm{drug}\;\mathrm{added}}{\mathrm{weight}\;\mathrm{of}\;\mathrm{oil}}\times 100\%\;\left(w|w\right)$$

### 2.9. Particle Size Stability of Nanoparticles at 4 °C and 37 °C

The physical stability of CUR MT NPs was evaluated during their storage at 4 °C for 6 months. The stability of blank MT NPs and CUR MT NPs was also analyzed at 37 °C in 10 mM PBS (pH 7.4) by adding 100 µL NPs to 13 mL PBS with a shaker at 150 rpm. At each time interval, an aliquot was removed and allowed to equilibrate to room temperature before measuring particle size.

### 2.10. Differential Scanning Calorimetry

The analysis was performed to determine the physical state of CUR using a DSC (Perkin Elmer 4000, Waltham, MA, USA). Blank MT NPs and CUR MT NPs were concentrated about 50-fold using Microcon Y-100 at 4 °C. The concentrated NPs were transferred to an aluminum pan and placed at room temperature for 48 h. The heating curves were recorded at a scan rate of 10 °C/min from 20 °C to 250 °C.

### 2.11. In Vitro Release Study

CUR release studies (*n* = 3) were completed at 37 °C using PBS as the release medium. CUR MT NPs (200 µL) was added into 20 mL phosphate buffered saline (PBS) and shaken at 135 rpm at 37 °C. At each time interval, 500 µL of sample was withdrawn. Released CUR was separated from CUR MT NPs using Microcon Y-100, and measured by HPLC as described above.

### 2.12. Lyophilization of CUR MT NPs

To determine the effect of lyophilization on the NPs, blank and CUR MT NPs were lyophilized using a freeze drier (VirTis Wizard 2.0, SP Industries, Gardiner, NY, USA). One milliliter of each sample was quickly frozen at −40 °C and then lyophilized using a program of 1 h at −10 °C for primary drying and 7 h at 15 °C for secondary drying at 200 mTorr. The resultant lyophilized product was reconstituted in 1 mL of D.I. water and shaken until the sample became homogeneous. The particle size, P.I., zeta potential, EE%, and DL% were measured as described above.

### 2.13. Cytotoxicity Studies

The cytotoxicity was tested in sensitive and resistant DU145 and PC3 cells using 3-(4,5-dimethylthiazol-2-yl)-2,5-diphenyltetrazolium bromide (MTT) assay. Cells were seeded into 96-well plates at 7000 cells/well and incubated at 37 °C for 24 h under 5% CO_2_. After overnight incubation, cells were treated with CUR, CUR MT NPs, and blank MT NPs at a concentration range from 0–270 µM. Due to low water solubility, we dissolved CUR in DMSO and controlled DMSO less than 3% in the treatment solutions. After 72 h of incubation, the medium was removed from each well and replaced with 90 µL of DMEM medium without phenol red and MTT (10 µL of 5 mg/mL in PBS). After incubating for 4 h at 37 °C with 5% CO_2_, 0.04 N HCl/isopropanol was added to each well and incubated for 5 min to dissolve formazan crystals formed by viable cells. The absorbance was measured at a test wavelength of 570 nm and a reference wavelength of 650 nm using the Synergy H1 Hybrid Multi-Mode Reader (BioTek, Winooski, VT, USA).

### 2.14. Statistical Analysis

Statistical comparisons were made with ANOVA, followed by pair-wise comparisons using Student’s *t* test using GraphPad Prism 6.0 (GraphPad Software, Inc., San Diego, CA). Results were considered significant at 95% confidence interval (*p* < 0.05).

## 3. Results

### 3.1. Selection of Oils and Surfactants and Preparation of CUR MT NPs

As shown in Table 1, CUR was found to have the highest solubility in Imwitor 900K among the oils. However, liquid oils could provide advantages over solid oils in terms of drug loading and NP stability [21]. The solubility of CUR in the tested surfactants was comparable. Hence, the results confirmed that the MT NP, composed of Miglyol 812 and TPGS, was optimal for CUR.

### 3.2. Solubility

The solubility of CUR in water and in the cell medium was 0.203 ± 0.053 µg/mL and 0.623 ± 0.031 µg/mL, respectively (*n* = 3).

### 3.3. Particle Size and Entrapment Efficiency

**Figure 1:**
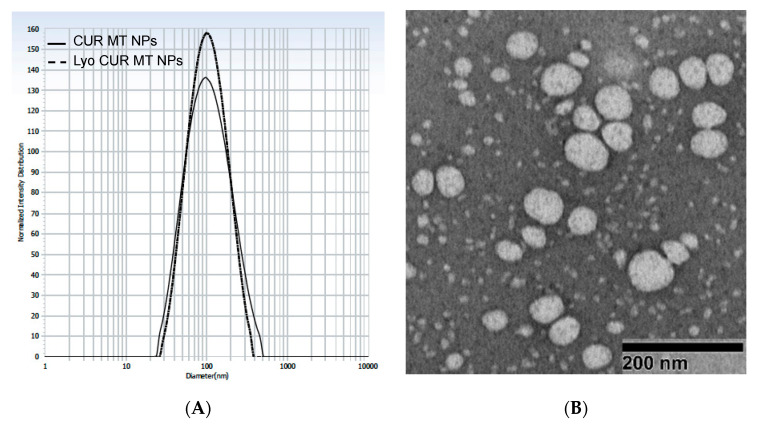
Particle size and size distribution of CUR MT NPs and Lyo CUR MT NPs. Particle size was measured by dynamic light scattering (CUR MT NPs and Lyo CUR MT NPs) (**A**) and TEM (CUR MT NPs) (**B**). Lyo CUR MT NPs were obtained by reconstitution of lyophilized CUR MT NPs.

As shown in Table 2 and Figure 1, particle size of CUR MT NPs was 138.9 nm with narrow size distribution (P.I. < 0.3), and zeta potential was −24.4 mV. The EE% and DL% of CUR MT NPs were 96.3 ± 6% (*n* = 3) and 3.6%, respectively. A TEM image confirmed the formation of spherical CUR MT NPs with particle size less than 100 nm.

### 3.4. Physical Stability

**Figure 2:**
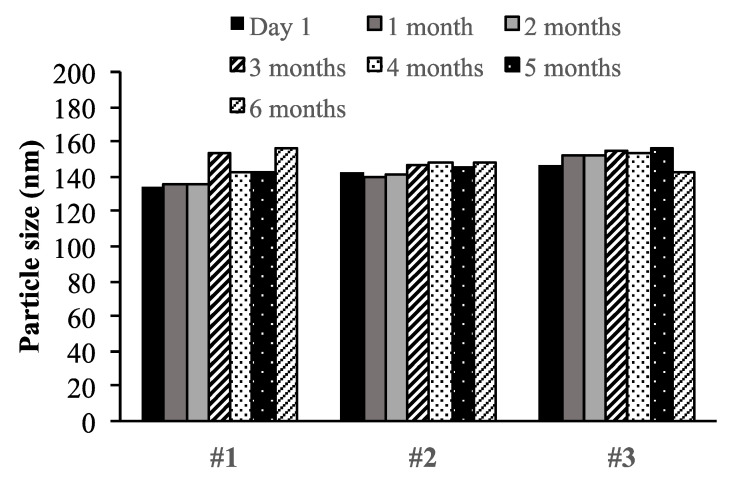
Long-term stability of CUR MT NPs stored at 4 °C. Three different batches of CUR MT NPs were monitored for particle size over 6 months. For all tested samples, P.I. < 0.25. Data are presented as the mean particle size of each batch. Particle size did not significantly change over the tested period (*p* > 0.05 for each batch).

**Figure 3:**
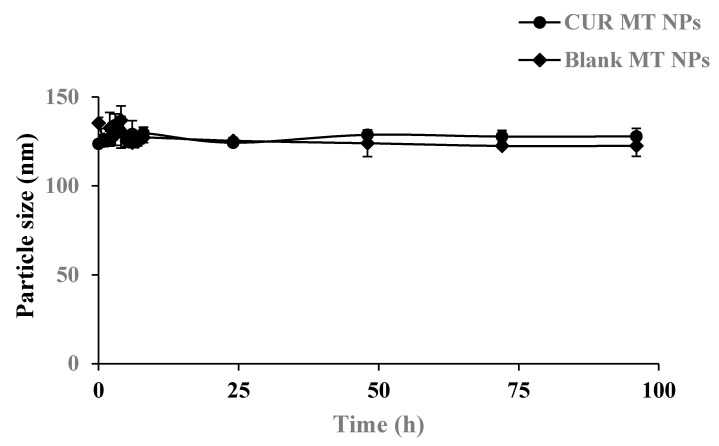
Physical stability of CUR MT NPs in a simulated physiological condition. The study was performed in PBS (pH 7.4) at 37 °C for up to 96 h. Blank MT NPs were used as a comparison. After equilibrating to room temperature, particle size was measured by dynamic light scattering. For all tested samples, P.I. < 0.25. Data are presented as the mean ± SD (*n* = 3). There was no significant difference in particle size over 96 h within the group or between the groups (*p* > 0.05).

The physical stability of blank MT NPs and CUR MT NPs was monitored at 4 °C over 6 months for long-term storage and at 37 °C in the PBS buffer for short-term stability in a simulated physiological condition. Both blank MT NPs and CUR MT NPs showed no significant changes in particle size at 4 °C over 6 months (Figure 2). Also, there were no significant changes in particle size for both blank MT NPs and CUR MT NPs incubated at 37 °C in PBS over 96 h (Figure 3).

### 3.5. Differential Scanning Calorimetry Analysis

**Figure 4:**
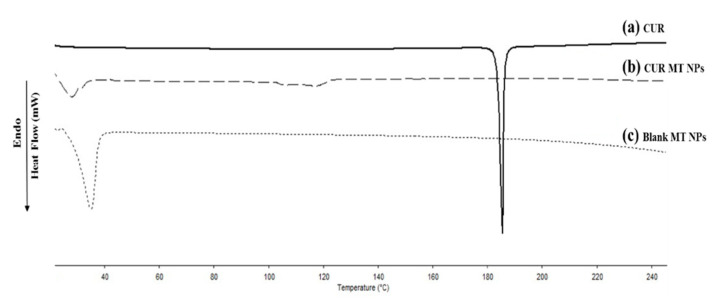
Differential scanning calorimetry (DSC) analysis of (**a**) CUR, (**b**) CUR MT NPs, and (**c**) blank MT NPs.

The melting peak of pure CUR was observed at 186 °C (Figure 4). Compared to pure CUR, there was no peak around 186 °C for CUR MT NPS, indicating that CUR was present as amorphous in the NPs. The melting endothermic peaks at 38 °C in both blank MT NPs and CUR MT NPs were the melting peak of TPGS.

### 3.6. In Vitro Release of Curcumin from the Nanoparticles

**Figure 5:**
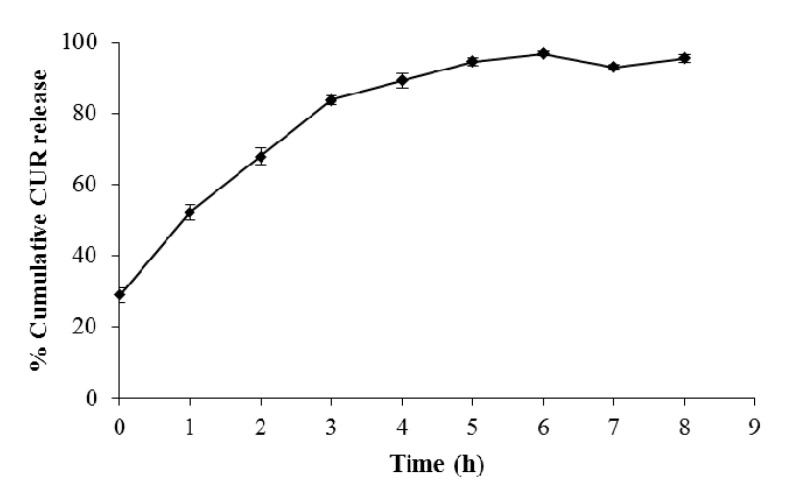
Release of CUR from CUR MT NPs in a simulated physiological condition. The study was performed in PBS (pH 7.4) at 37 °C for 8 h. Released CUR was separated from CUR MT NPs by Microcon-Y100 and measured by HPLC. Data are presented as the mean ± SD (*n* = 3).

The in vitro release profile of CUR MT NPs was tested in PBS at 37 °C. The cumulative CUR release from CUR MT NPs is shown in Figure 5. CUR showed a burst release from CUR MT NPs at the beginning and then continually released over 5 h.

### 3.7. Lyophilization

The rehydrated lyophilized CUR MT NPs (Lyo CUR MT NPs) had 93.1% of EE% and 3.5% of DL%. As shown in Figure 1, CUR MT NPs and Lyo CUR MT NPs had similar particle size and size distribution. Based on statistical analysis, lyophilization did not change the physio-chemical properties of CUR MT NPs, including particle size, P.I., zeta potential, EE%, and DL% (*p* > 0.05) (Table 2 and Figure 1).

### 3.8. In Vitro Cytotoxicity Studies

**Figure 6:**
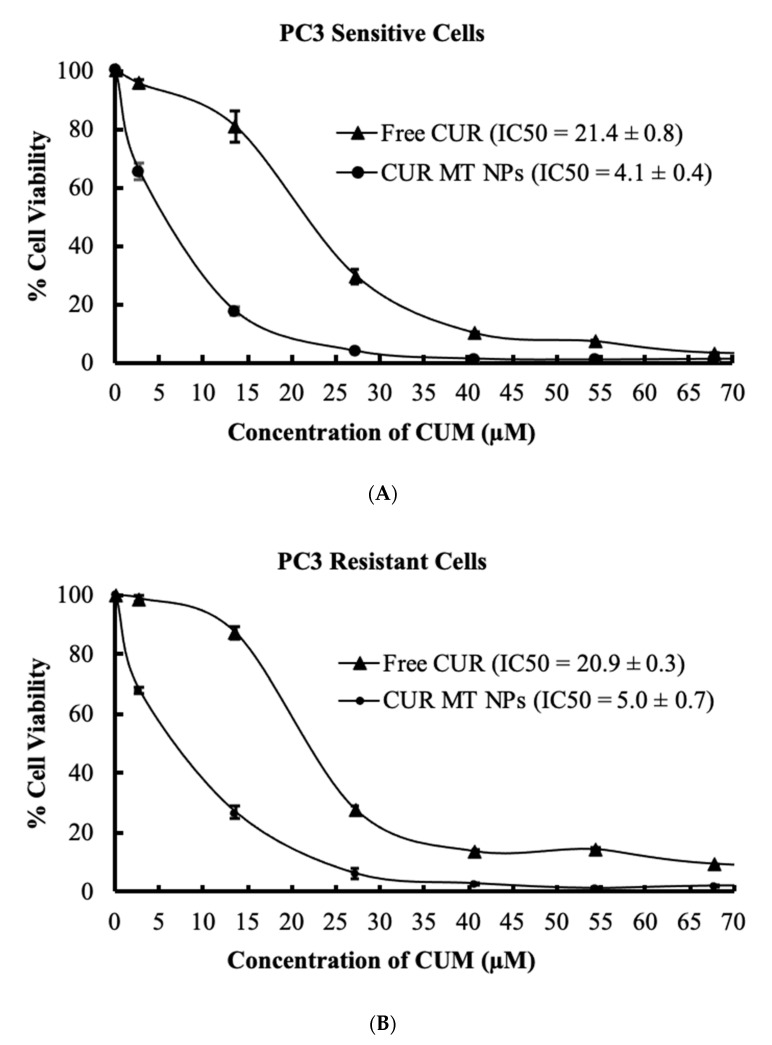

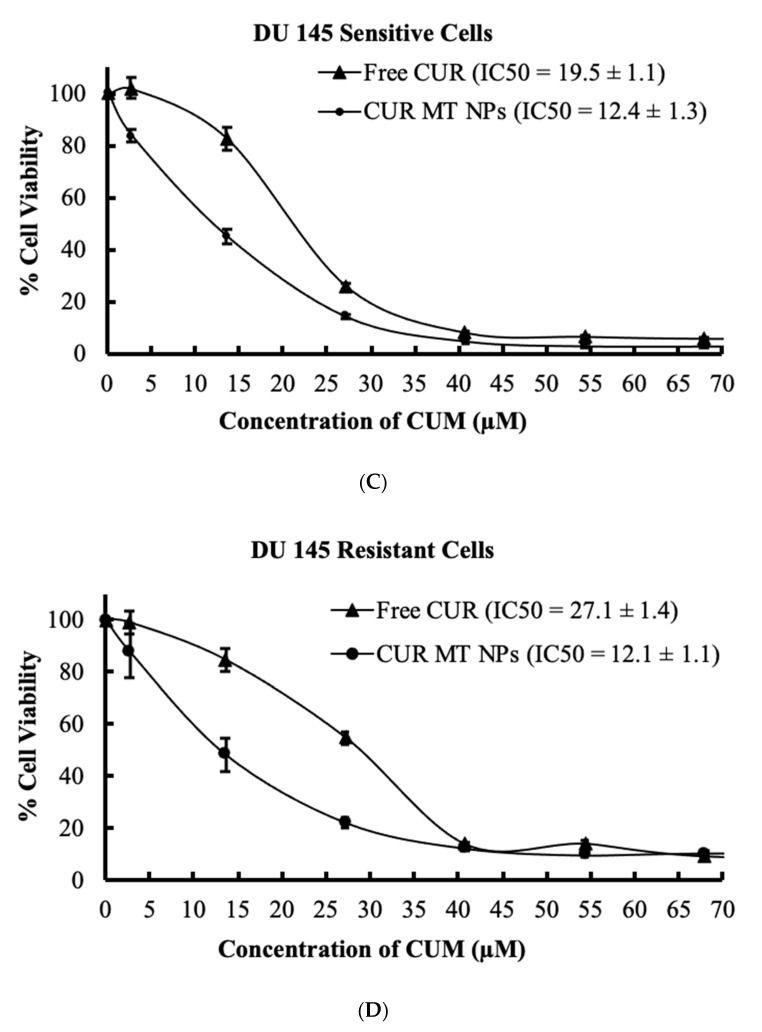
Cytotoxicity studies of CUR and CUR MT NPs in castration-resistant prostate cancer (CRPC) cells. (**A**) DTX-sensitive PC3 cells, (**B**) DTX-resistant PC3 cells, (**C**) DTX-sensitive DU145 cells, and (**D**) DTX-resistant DU145 cells. Data are presented at the mean of three independent measurements (*n* = 3) ± SD. Cells were treated for 72 h with samples. Cell viability was measured by 3-(4,5-dimethylthiazol-2-yl)-2,5-diphenyltetrazolium bromide (MTT) assay.

**Figure 7:**
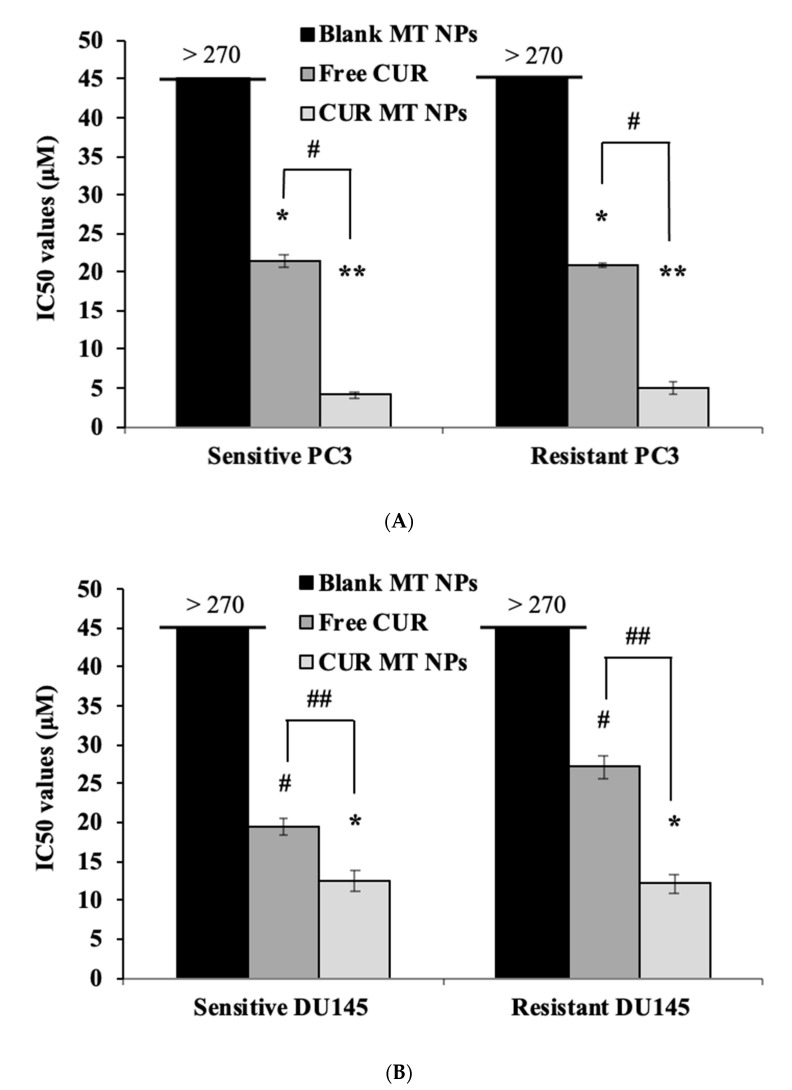
Comparison of IC_50_s for CUR, CUR MT NPs, and blank MT NPs in CRPC cells. (**A**) IC_50_ values (μM) of free CUR and CUR MT NPS in DTX-sensitive and -resistant PC3 cells. (**B**) IC_50_ values (μM) of free CUR and CUR MT NPS in DTX-sensitive and -resistant DU145 cells. Data are presented at the mean of three independent measurements (*n* = 3) ± SD. #, ## *p* < 0.05 within the compared treatments; *, ** *p* > 0.05 within the compared treatments.

Figure 6 shows the results of cytotoxicity studies of CUR and CUR MT NPs in sensitive and resistant PC3 cells and sensitive and resistant DU145 cells, and Figure 7 shows the statistical comparison of the IC_50_s of these treatments. As shown in Figure 7A, free CUR showed the same cytotoxicity in sensitive and resistant of PC3 cell lines (*p* > 0.05). In contrast, the IC_50_ value of free CUR was 1.4-fold higher in resistant DU145 than that in sensitive DU145 (Figure 7B). Compared to free CUR, CUR MT NP decreased the IC_50_ value over 5-fold in both sensitive and resistant PC3 cell lines and about 2-fold in both sensitive and resistant DU145 cell lines (Figure 7A,B). Importantly, CUR MT NPs showed no significant differences in the IC_50_ values in sensitive and their matching resistant cells for both PC3 and DU145 (Figure 7A,B) (*p* > 0.05). The IC_50_s of blank MT NPs were higher than 270 µM in the tested cells, indicating low cytotoxicity of blank MT NPs.

## 4. Discussion

CUR has been studied in many clinical trials for various cancers with little success although CUR exhibits strong activity for cancer prevention in animals. It is well known that CUR has low solubility; however, no study to date has reported water solubility data for CUR. According to our measurement, solubility of CUR in water is only 0.202 µg/mL. Moreover, solubility of CUR increased over 3-fold in the cell medium compared to water, demonstrating that the composition of the solution has great influence on CUR solubility. It is possible that the variation of CUR solubility in different environments could cause controversial results from lab studies and human studies. On the other hand, the poor solubility confirmed that CUR must be formulated in such a way as to overcome the solubility issues in order to apply its therapeutic effect.

We developed CUR MT NPs in this study. Our CUR NPs demonstrated stability at 4 °C over 6 months (Figure 2) and at 37 °C over 96 h (Figure 3). Moreover, CUR MT NPs are lyophilizable without losing their properties, which grants the other opportunity for long-term storage (Table 2 and Figure 1). Liquid lipid Miglyol 812 reduces particle crystallinity, conferring better stability and suitability than solid lipids [22]. The optimal 6-month stability of CUR MT NPs could result from the use of liquid Miglyol 812 in the NPs. CUR MT NPs were smaller than 140 nm with narrow size distribution (Figure 1). Because NPs tend to aggregate during long-term storage, lyophilization is a promising method to remove water and keep NPs in dry states for long-term storage. However, lyophilization often causes an increase of particle size even with cryoprotectants. Interestingly, CUR MT NPs can be lyophilized without using cryoprotectants. After reconstitution, properties of lyophilized CUR MT NPs, including particle size, size distribution, DL and EE, did not change, which shows the potential of developing CUR MT NPs as a medical injection. Studies have suggested that particles between 100 to 200 nm are desired for adhesion to and interaction with cells as well as taking advantage of the EPR effect [23]. Thus, our particle size is desirable for cancer drug delivery. According to the DSC study, CUR dissolved in Miglyol 812 and TPGS during the NP preparation and existed as amorphous in the NPs (Figure 4). CUR MT NPs showed a relatively rapid, but complete release over 5 h (Figure 5), demonstrating that NPs enhanced the solubility of CUR and could facilitate the solubilization of CUR in the blood.

CUR has pleiotropic properties that modulate numerous targets including proteins (i.e., tubulin), transcription factors, growth factors and their receptors, cytokines, enzymes, and apoptosis [24,25]. Thus, CUR has been used for cancer therapy and prevention, and also as a chemosensitizer to combine with other drugs (e.g., DTX) to overcome drug resistance in various cancers [26]. However, there have been no studies of CUR itself as a therapeutic drug in DTX-resistant CRPC. Compelling evidence showed that NPs could overcome P-gp-mediated drug resistance in cancers [27,28,29]. In our previous studies, we demonstrated that the blank MT NPs inhibited P-gp function [20]. Thus, by combining multi-targeted behaviors and a wide spectrum of actions of CUR with the advantages of the MT NPs, we hypothesized that CUR MT NPs could be potentially used as a therapeutic drug to overcome DTX-resistant CRPC. It is known that the resistance in DU145 and PC3 cells is controlled by different resistant mechanisms [7]. Overexpression of P-gp has been identified as the only main cause of resistance in DU145 cells, but P-gp does not play a role in resistant PC3 cells [8]. Alterations of microtubule formation, down-regulation of microtubule-related genes, alterations in the expression of tubulin composition, and up-regulation of apoptosis genes were identified in the resistant PC3 cells [7]. To test our hypothesis, we studied the cytotoxicity of CUR MT NPs in both resistant and sensitive DU145 and PC3 cell lines.

Our data showed that free CUR had the same IC_50_s in both sensitive and resistant PC3 cells, indicating that. in spite of resistance to DTX, PC3-resistant cells were not resistant to free CUR (Figure 7A). In contrast, the IC_50_ of free CUR in DU145-resistant cells was higher than that in DU145-sensitive cells, indicating, in addition to resistance to DTX, DU145-resistant cells are also resistant to CUR (Figure 7B). According to the results, CUR MT NPs were 5-fold more potent than free CUR in PC3 cells lines and about 2-fold more potent than free CUR in DU145 cells, demonstrating that the NPs enhanced CUR cytotoxicity in these cancer cells (both sensitive and resistant cells). Importantly, the IC_50_s of CUR MT NPs did not have significant differences between sensitive and resistant PC3 cells (4.1 ± 0.4 µM vs. 5.0 ± 0.7 µM, *p* > 0.05) or between sensitive and resistant DU145 cells (12.4 ± 1.3 µM vs. 12.1 ± 1.1 µM, *p* > 0.05) (Figure 7A,B), which demonstrated that CUR MT NPs completely overcame the resistance not only in PC3 cells but also in DU145 cells. The results demonstrate that the combination of CUR (a multi-targeted agent) and MT NP (a drug-carrier inhibiting P-gp) really synergize their advantages and provide a novel therapeutic strategy for the treatment of DTX-resistant CRPC. Considering that CUR is a relatively safe compound, CUR MT NPs with the IC_50_s around 10 µM will be an effective therapeutic drug for the treatment of DTX-resistant CRPC.

Further cell studies (e.g., cell uptake studies, apoptosis, and the state of autophagy) are expected to understand the underlying mechanisms, and animal studies are granted to demonstrate the therapeutic efficacy of CUR MT NPs. To the best of our knowledge, this is the first report to use CUR NPs for the treatment of DTX-resistant CRPC. This study also demonstrates the therapeutic potential of CUR for the treatment of other drug-resistant cancers.

## 5. Conclusions

Taken together, we are the first to demonstrate that CUR MT NPs have potential to overcome DTX-resistant CRPC. We successfully developed and characterized CUR MT NP with high DL and high EE. CUR MT NPs were stable at 4 °C over 6 months. They could be lyophilized without using cryoprotectants. In addition to increasing the potency of CUR in sensitive cancer cells, CUR MT NPs completely overcame resistance and restored CUR potency in both resistant PC3 and DU145 cells. By integrating the advantages of CUR and MT NPs, CUR MT NPs have potential as a novel therapeutic drug to overcome DTX resistance in CRPC.

## Figures and Tables

**Table 1:** Solubility of curcumin in oils and surfactants.

Oil	Solubility (*w*/*w*)	Surfactant	Solubility (*w*/*w*)
Miglyol 812	0.0153	Tween 20	0.166
Imwitor 491	0.0261	Tween 80	0.141
Kollisolv MCT 70	0.0179	Labrasol	0.173
Labrafac	0.0120	Poloxamer 188	0.153
Imwitor 900K	0.0512	TPGS	0.164
Imwitor 960	0.0488	Gelucire 44/14	0.153
Compritol 888	0.0160		

Note: *w*/*w* = weight by weight (gram/gram).

**Table 2:** Physico-chemical properties of CUR MT NPs (*n* = 3).

Formulations	CUR (µg/mL)	Particle size ^a^ (nm) *	P.I. *	Zeta Potential (mV) *	EE% *	DL% *
CUR MT NPs	150	138.7 ± 5.4	0.268 ± 0.033	−24.4 ± 3.3	96.3 ± 6.0	3.6
Blank MT NPs	n/a	119.9 ± 3.5	0.246 ± 0.016	−20.6 ± 1.9	n/a	n/a
Lyo CUR MT NPs	150	101.3 ± 11.1	0.268 ± 0.033	−16.6 ± 3.7	93.1 ± 5.8	3.5

^a^ The data are presented as the mean particle size of NPs in different batches ± SD (*n* = 3). * *p* > 0.05 when CUR MT NPs and lyophilized CUR MT NPs (Lyo CUR MT NPs) for each labeled parameter are compared.
